# Type 2 Diabetes From the Perspective of Telomere Biology

**DOI:** 10.1002/edm2.70137

**Published:** 2026-03-23

**Authors:** Hongye Cao, Haixia Feng, Zhen Shen, Yanqin Huang

**Affiliations:** ^1^ The First Clinical Medical College Shandong University of Traditional Chinese Medicine Jinan China; ^2^ Department of Endocrinology Shandong Provincial Hospital of Traditional Chinese Medicine Jinan China

**Keywords:** T2DM, telomerase, telomere

## Abstract

**Background:**

Type 2 diabetes mellitus (T2DM) has emerged as one of the most critical public health challenges worldwide, with its prevalence continuing to rise steadily. Current projections estimate that the global number of individuals affected by T2DM will reach approximately 783 million by 2045, imposing an increasingly severe burden on healthcare systems globally. In recent years, growing attention has been directed toward the relationship between telomere biology and metabolic disorders. Telomeres, the protective structures at the ends of chromosomes, serve as key markers of cellular aging, with their length and integrity reflecting biological age. Telomerase, a specialised enzyme complex, plays a central role in maintaining telomere stability. Accumulating evidence suggests that telomere shortening and dysfunction not only accelerate organismal aging but may also contribute to the pathogenesis of T2DM through multiple molecular pathways.

**Objective:**

This review aims to systematically synthesise recent research advances regarding the association between telomeres and T2DM, and to explore the clinical translational potential of telomere length and telomerase activity as biomarkers of disease risk or as potential therapeutic targets.

**Methods:**

A systematic search of relevant articles was conducted across databases including PubMed, Web of Science, and CNKI. Search terms comprised ‘Telomere’, ‘Telomerase’, ‘T2DM’ and ‘Treatment’. Through screening and integration of evidence from cellular experiments, animal model studies, population‐based epidemiological investigations and clinical observational research, this review systematically synthesises the mechanistic evidence and current research landscape regarding the association between telomeres and T2DM.

**Results:**

Accumulating evidence consistently indicates a significant association between telomere biology and the development and progression of T2DM. Key mechanisms primarily involve oxidative stress and cellular apoptosis, while telomere dysfunction may also contribute to glucose metabolism disorders by impairing pancreatic β‐cell function and peripheral tissue insulin sensitivity. Dynamic changes in telomeres hold promise as novel biomarkers for assessing T2DM risk and disease progression.

**Conclusions:**

A close and bidirectional relationship exists between telomere biology and T2DM. Deeper insights into this association not only enhance our understanding of T2DM pathogenesis but also offer potential therapeutic targets for anti‐aging‐based interventions, highlighting important clinical translation prospects. Future research should further clarify the causal relationships involved and explore strategies aimed at preserving telomeres or modulating telomerase activity to delay diabetes progression and prevent its complications, thereby advancing novel approaches for early risk assessment and precision management of T2DM.

## Graphical abstract

This graphical abstract illustrates the bidirectional relationship between the telomere/telomerase system and T2DM: telomere attrition contributes to pancreatic β‐cell dysfunction and insulin resistance through mechanisms such as oxidative stress and apoptosis, while T2DM‐related hyperglycemia and chronic inflammation further exacerbate telomere damage. This cyclic interaction not only deepens the understanding of disease pathogenesis but also highlights the translational potential of telomere biology in providing novel directions for the precision prevention and management of T2DM.

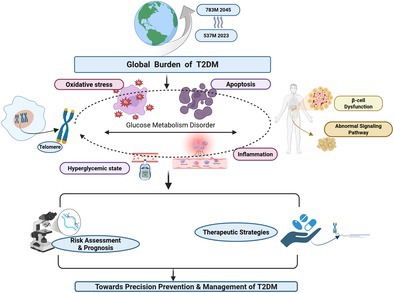

## 1 Introduction

T2DM is a chronic metabolic disease that arises from an interplay of genetic susceptibility and unhealthy lifestyle choices, and is mainly defined by sustained high blood glucose levels. The underlying disease mechanisms are intricate and involve multiple factors, including the development of insulin resistance and the gradual impairment of pancreatic β‐cell function. T2DM is increasingly becoming a significant concern for public health, both in China and globally. In addition to its effects on personal health, the condition can give rise to a variety of systemic complications that affect the vascular and nervous systems, potentially harming critical organs such as the heart, brain, kidneys, eyes, lower extremities, and the nervous system [1, 2, 3]. Currently, the primary approach to managing T2DM focuses on controlling blood sugar levels. Although these strategies can extend life expectancy, there is still a lack of definitive and curative treatments. As a result, preserving and restoring pancreatic β‐cell function has emerged as a key objective in the therapeutic management of T2DM [4].

Telomeres, as protective structures at the ends of chromosomes, gradually shorten with each cell division. When telomeres shorten to a critical length, they trigger the cell senescence or apoptosis program. Therefore, telomere length is a key indicator reflecting the replicative senescence state of cells [5, 6]. This mechanism of replicative senescence driven by telomere shortening is precisely the molecular basis for the upper limit of the number of cell divisions (i.e., the ‘Hayflick limit’) that normal human somatic cells have when cultured in vitro [7]. A range of clinical investigations has revealed that telomeres in white blood cells and pancreatic islet β‐cells of individuals with T2DM are notably shorter compared to those in metabolically healthy individuals [8]. Furthermore, telomere length has been found to negatively correlate with the duration of diabetes and the degree of glycemic control [8, 9, 10]. Under normal conditions, pancreatic islet β‐cells, as terminally differentiated cells, exhibit silenced telomerase expression [11]. This inherent deficiency in telomerase activity limits their capacity to repair telomeric damage effectively, thereby increasing susceptibility to telomere shortening‐induced activation of the p53‐dependent apoptotic pathway [12]. In T2DM, metabolic stress such as high blood sugar accelerates telomere attrition, further amplifying β‐cell apoptosis and becoming a key mechanism in the occurrence and development of diabetes [13].

Telomerase is a ribonucleoprotein enzyme complex that is essential for cellular proliferation and division. Its main function involves the synthesis of telomeric DNA repeats, which helps offset the progressive telomere attrition that occurs during cell division, thereby preserving chromosomal integrity and extending the cell's replicative lifespan. Telomerase plays an important role in maintaining genomic stability, but its activity is by no means ubiquitous in all physiological processes. On the contrary, the expression of telomerase is strictly regulated at multiple levels [14]. This is a core component of the evolutionarily conserved anti‐cancer barrier. The progressive attrition of telomeres can effectively prevent the unlimited proliferation of somatic cells [15]. In most differentiated somatic cells, the catalytic subunit telomerase reverse transcriptase (TERT) gene is in an epigenetically silenced state. Only in specific cell types that require proliferative potential (such as stem cells, germ cells and activated lymphocytes), through signalling pathways such as the Wnt protein/beta‐catenin signalling pathway, the enrichment of Histone H3 lysine 4 trimethylation (H3K4me3) modification is induced and the chromatin conformation is remodelled, allowing activators such as the cellular myelocytomatosis oncogene (c‐Myc) to bind [16]. This highly restricted regulatory pattern essentially uses the telomere attrition mechanism as a natural defence against cancer by limiting telomerase expression [17].

In T2DM, insulin resistance frequently triggers enhanced lipolysis and dysregulation of lipid metabolism, leading to a marked elevation in circulating free fatty acid (FFA) levels [18]. Evidence indicates that the characteristic hyperglycemic and high‐FFA milieu acts synergistically to promote excessive reactive oxygen species (ROS) accumulation. This oxidative stress exerts dual detrimental effects: first, it directly induces oxidative damage to telomeric DNA, exemplified by the formation of 8‐oxoguanine [19]; second, it activates DNA methyltransferase DNA‐methyltransferase 3 beta (DNMT3B), which targets the Cytosine‐phosphate‐Guanine (CpG) island in the core region of the TERT promoter (from −200 bp to the transcription start site), catalysing its hypermethylation. This epigenetic modification impedes the binding of key transcription factors such as c‐Myc and Upstream Stimulatory Factor 1 (USF1), resulting in transcriptional repression of TERT and consequent loss of telomerase activity. However, this metabolic stress‐induced silencing of TERT represents only a partial manifestation of broader epigenetic complexity. Methylation regulation of the TERT promoter exhibits pronounced regional specificity and contextual dependency. In somatic cells—including diabetic vascular endothelial cells—hypermethylation of the promoter's core region promotes gene silencing, accelerates telomere shortening and dysfunction, and ultimately contributes to impaired NO biosynthesis, increased vascular permeability, and basement membrane thickening. These alterations collectively exacerbate microvascular complications in T2DM and promote instability of atherosclerotic plaques in large vessels, thereby intensifying the overall diabetic pathology [20].

Notably, cancer cells exhibit a pattern of hypomethylation in the core region (particularly within the CpG island shore) accompanied by TERT reactivation. Paradoxically, hypermethylation in distal regulatory regions (e.g., from −2.6 to −3.3 kb upstream) leads to the displacement of repressive factors such as Methyl‐CpG‐binding Protein 2 (MECP2) and CCCTC‐Binding Factor (CTCF), thereby reshaping the three‐dimensional chromatin architecture and synergistically promoting TERT transcription. This bidirectional regulation demonstrates that the transcriptional fate of TERT is determined not by a simple inverse correlation between methylation and gene activity, but rather by the spatial positioning of methylation sites (repressive in the core, activating in distal regions), higher‐order chromatin topology, and contextual signals from the tissue microenvironment—including metabolic stress in diabetes or oncogenic stimuli [21].

The relationship among telomeres, telomerase, and T2DM has attracted growing attention in recent research. This paper seeks to explore the involvement of telomeres and telomerase in the development and progression of T2DM, clarify the mechanisms behind their actions, and assess their potential application as novel therapeutic targets.

## 2 Overview of Telomere and Telomerase Biology

Telomeres, as functional cap‐like structures at the ends of eukaryotic chromosomes, have their integrity precisely maintained by the shelterin complex. This protein complex, composed of subunits including Telomeric Repeat‐binding Factor 1 (TRF1), Telomeric Repeat‐binding Factor 2 (TRF2), and Repressor/Activator Protein 1 (RAP1), protects chromosome ends by facilitating the formation of a higher‐order T‐loop structure, which prevents telomeric DNA from being erroneously recognised as double‐strand breaks and thus ensures genomic stability [22]. Telomere length is dynamically regulated through a balance between telomerase‐mediated elongation and replication‐dependent shortening. Once this balance is disrupted by factors, such as metabolic stress, it will trigger a continuous DNA damage response (DDR), ultimately inducing cellular senescence or apoptosis through p53‐dependent or ‐independent pathways [23]. Consequently, telomeres serve not only as guardians of genomic integrity but also as molecular signalling hubs that integrate intrinsic and extrinsic cues to modulate cell fate decisions. Their dysfunction is strongly implicated in aging‐associated pathological processes [24]. The length of telomeres is modulated by an interplay of biological mechanisms (such as cellular proliferation), hereditary influences (including the activity of genes involved in telomere maintenance), and external stressors (like inflammation and oxidative stress) [25]. Studies suggest that telomeres progressively shorten at a steady pace as people grow older. This inherent biological process can be further accelerated by the development of specific diseases, with T2DM being a notable example [26]. High levels of blood glucose contribute to oxidative stress and activate DNA damage mechanisms, which in turn negatively affect telomere integrity and accelerate their erosion. At the same time, the deterioration of telomeres worsens glucose metabolism by reducing the ability of pancreatic islet β‐cells to regenerate and secrete insulin, leading to a self‐reinforcing, two‐way detrimental loop [27, 28].

Telomerase consists mainly of two essential components: the telomerase RNA component (TERC) and TERT [29]. Its main role is to use its RNA as a template for reverse transcription, synthesising telomeric DNA repeats, which helps preserve and restore telomere length [30]. As a critical RNA component of telomerase, TERC contains the template sequence 5′‐CAACCCCAA‐3′, which directs the TERT catalytic subunit to generate TTAGGG repeats at telomeric ends. By forming evolutionarily conserved pseudoknot and stem‐loop structures, TERC contributes to the structural stabilisation of TERT, boosts its enzymatic efficiency, and enables precise localization of the telomerase complex to chromosome termini. Consequently, TERC functions not only as a genetic blueprint for telomere synthesis but also as a central regulatory platform [31]. In the absence of TERC, TERT is unable to perform template‐directed reverse transcription, resulting in total loss of telomerase function [32]. As the catalytic reverse transcriptase subunit of telomerase, TERT directly utilises the RNA template of TERC to synthesise telomeric repeat sequences. Its active centre catalyses nucleotide polymerisation through the conserved RT domain. At the same time, it relies on the TEN domains to anchor the 3′ end of telomeric DNA to ensure the continuous extension of the repeat sequences. Thus, TERT serves as the principal effector in telomere lengthening and cellular immortalization [33]. Without TERT, telomerase cannot carry out catalysis, leading to failure in preserving telomere integrity and equilibrium [29].

Recent research has shown that TERT, in addition to its well‐established role in telomere regulation, engages in various non‐traditional activities. One notable example is its ability to move into mitochondria and perform specific functions within these cellular compartments. However, the precise biological implications and the molecular mechanisms driving TERT's mitochondrial localization are still under active investigation and remain controversial. Some studies have demonstrated that under mild oxidative stress conditions, such as exposure to low‐dose Hydrogen Peroxide (H_2_O_2_), mitochondrial translocation of TERT enhances mitochondrial complex I activity and scavenges ROS, thereby exerting a protective effect on mitochondrial DNA [34, 35, 36]. In contrast, under high‐glucose stress, human telomerase reverse transcriptase (hTERT) translocates to mitochondria and aberrantly binds to the mitochondrial RNA component RNA Component of Mitochondrial RNA Processing Endoribonuclease (RMRP). This interaction forms a complex that directly inhibits the activity of the mitochondrial DNA polymerase DNA Polymerase Gamma (POLG), resulting in impaired Mitochondrial DNA (mtDNA) replication and the accumulation of deletion mutations. Concurrently, the hTERT‐RMRP complex interferes with the binding of the transcription factor Mitochondrial Transcription Factor A (TFAM) to mtDNA, compromising mitochondrial DNA repair capacity. Ultimately, this cascade amplifies oxidative damage to mtDNA caused by free radicals and promotes cellular apoptosis [37, 38]. The essence of this controversy is that the mitochondrial function of TERT is environmentally dependent, and its ultimate effect as a redox regulator may depend on factors such as local ROS concentration, stress duration, and cell metabolic state. This provides new ideas for intervention strategies targeting the subcellular localization of TERT in the context of metabolic diseases such as diabetes.

## 3 Telomere and Telomerase: Biology and Their Role in the Pathogenesis of T2DM

### 3.1 Oxidative Stress

Metabolic disorders, including T2DM, are strongly correlated with increased levels of oxidative stress within the body [39, 40]. This is associated with variations in the regulation of uncoupling protein 2 (UCP2) gene expression [8]. UCP2 functions as a possible negative regulator of ROS generation by uncoupling oxidative phosphorylation, thereby reducing the electrochemical potential across the mitochondrial inner membrane [41]. When UCP2 expression is downregulated, ROS production increases [42], further intensifying oxidative stress [43]. The buildup of internal oxidative stress can trigger the translocation of TERT from the nucleus to the cytoplasm, ultimately diminishing total telomerase activity, especially in pancreatic β‐cells [44, 45].

**FIGURE 1:**
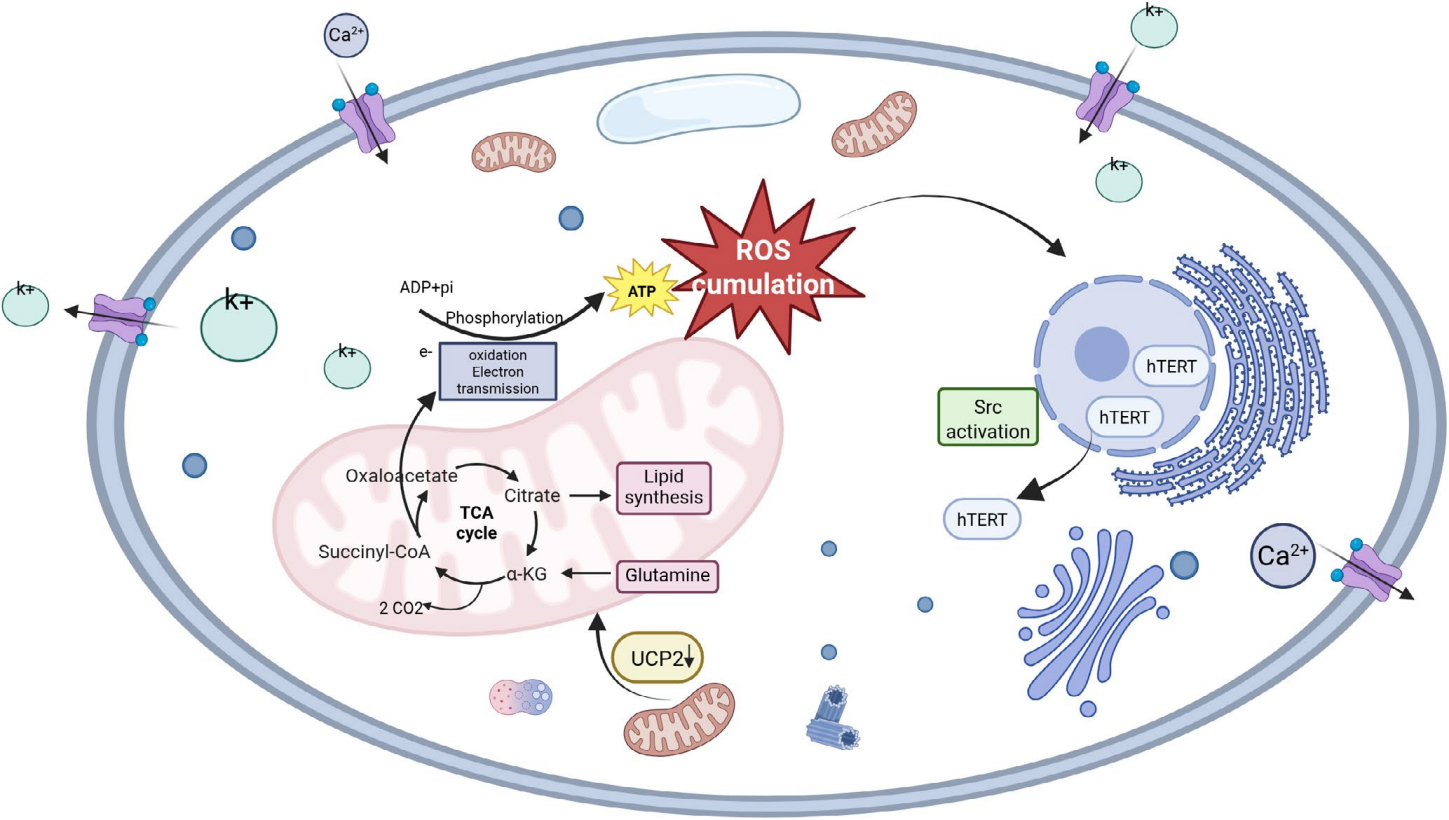
Oxidative stress. Core circuit of mitochondrial‐hTERT interplay driving oxidative stress under metabolic stress. Hyperglycemia/lipotoxicity → mitochondrial dysfunction (TCA cycle flux perturbation, UCP2↓ → ETC electron leakage) → ROS burst → Src kinase activation → Src‐dependent hTERT nucleo‐cytoplasmic shuttling → decreased mitochondrial hTERT and Nrf2 suppression → impaired ROS scavenging and Ca^2+^ dyshomeostasis → sustained ‘metabolic memory’ cycle, culminating in insulin secretion defects in T2DM (Key: ATP, adenosine triphosphate; ETC, electron transport chain; TCA, tricarboxylic Acid Cycle).

Telomere attrition contributes to the worsening of T2DM pathology. It triggers a DNA damage response, activates the p53/p21 signalling cascade, and pushes pancreatic β‐cells into a state of replicative senescence. Research using telomerase‐deficient mouse models has shown that a 60% decline in the ability of β‐cells to proliferate results in compromised glucose‐stimulated insulin secretion, which eventually leads to reduced glucose tolerance [46]. Moreover, findings suggest that damage to telomeres stimulates the secretion of pro‐inflammatory cytokines, such as interleukin‐6 (IL‐6) and tumour necrosis factor‐α (TNF‐α). These signalling molecules then prompt the activation of Nicotinamide Adenine Dinucleotide Phosphate (NADPH) oxidase, increase local concentrations of ROS, and create a self‐sustaining loop of ‘oxidative stress–telomere erosion–inflammatory reaction’, further hastening the onset and progression of T2DM [47]. The relevant mechanism described above is illustrated in Figure 1.

### 3.2 Apoptosis

TERT is capable of suppressing cell apoptosis through the regulation of telomerase activity and facilitating telomere extension [48, 49]. Normally, the expression of TERT is kept under tight control. However, in certain conditions, this inhibition is lifted, leading to elevated TERT expression. As a result, telomerase becomes active, which either halts or reduces the rate of telomere shortening and, in some cases, may even induce telomere lengthening [50]. For example, in certain malignancies including melanoma, bladder cancer, hepatocellular carcinoma and glioma, TERT upregulation is primarily driven by high‐frequency somatic mutations in its promoter region, notably the recurrent hotspots C228T (chr5:1,295,228 C>T) and C250T (chr5:1,295,250 C>T). These mutations create new ETS transcription factor binding sites (such as the binding site of GABP), thereby enhancing TERT transcription and telomerase activation. This phenomenon has been widely confirmed in a variety of cancer types mentioned above. Among them, the somatic mutation rate of bladder cancer is as high as 74% [51]. Notably, germline variants in the TERT promoter (distinct from somatic mutations) are documented in rare hereditary cancer syndromes, including familial melanoma, conferring elevated cancer risk. Among familial melanoma patients with germline variants, somatic TERT promoter mutations arise in ~74% of tumours [52]. However, the incidence of TERT promoter germline mutations in the population is extremely low and is not the main pathway for TERT activation in sporadic tumours. Beyond genomic alterations, transcriptional pathway dysregulation critically drives TERT activation. In non‐small cell lung cancer (NSCLC), c‐Myc overexpression elevates TERT mRNA ≥ 3‐fold and telomerase activity through direct binding to canonical in the TERT promoter. This is frequently linked to PI3K/AKT/mTOR hyperactivation, which stabilises c‐Myc and counteracts epigenetic repression (e.g., DNMT3B‐mediated methylation), enabling sustained TERT expression independent of promoter mutations [53]. Regardless of whether it is through promoter mutations or abnormal transcription factor pathways, the abnormal upregulation of TERT can relieve the restriction of cellular replicative senescence, endow the ability of immortalization, provide a necessary prerequisite for the continuous accumulation of carcinogenic mutations, and ultimately synergistically drive malignant transformation [54].

**FIGURE 2:**
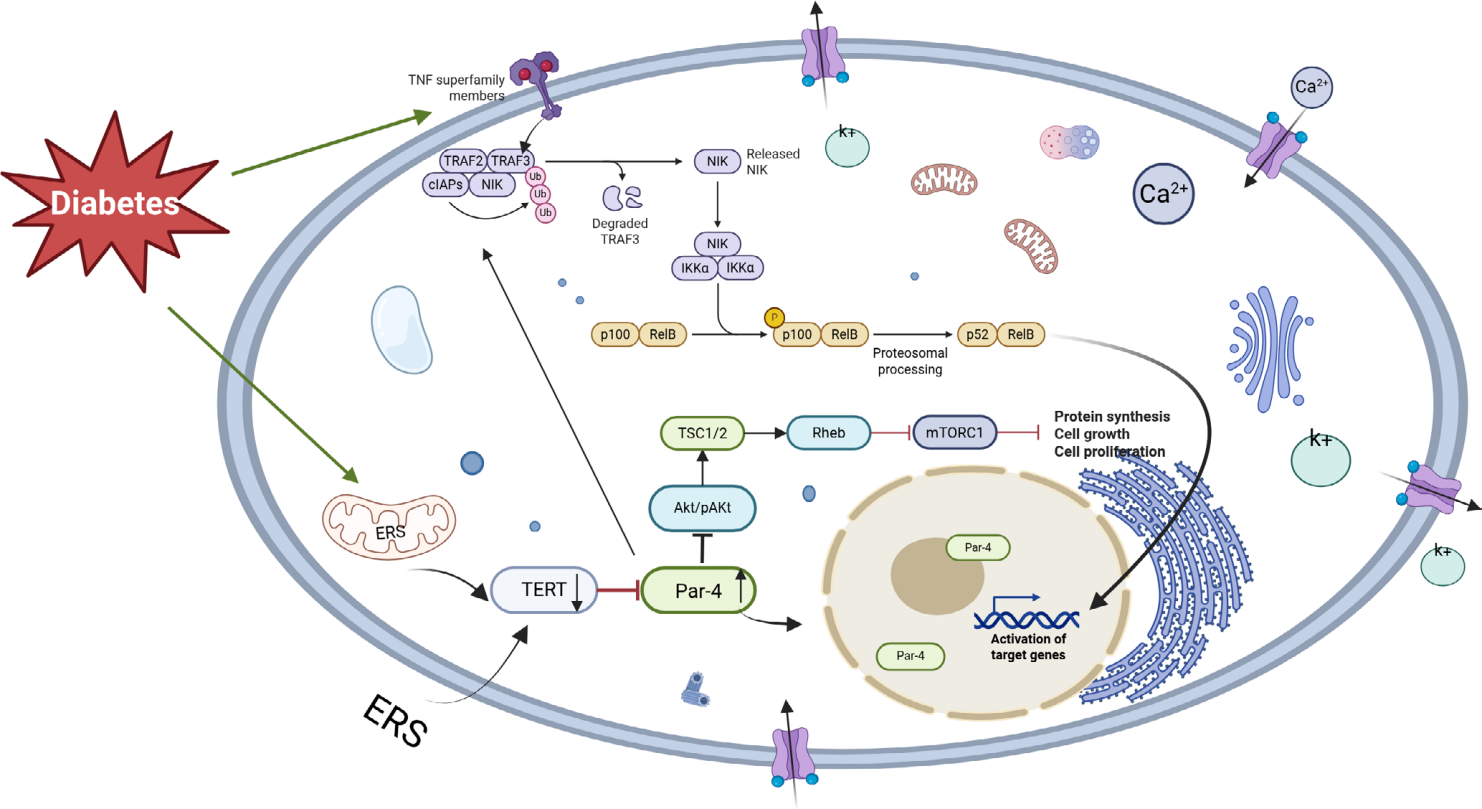
Apoptosis. Integrated regulation of telomeres, telomerase, and apoptosis in T2DM. Endoplasmic reticulum stress (ERS) induced by hyperglycemia/lipotoxicity suppresses TERT activity, triggering the pro‐apoptotic factor Par‐4 upregulation and nuclear translocation. This activates downstream apoptotic targets (e.g., FADD/caspase‐8). ERS synergizes with dysregulated Akt/mTORC1 (cell growth suppression) and non‐canonical NF‐κB signalling (TNF‐superfamily → TRAF2/3 → NIK → p52/RelB), collectively driving telomerase dysfunction–apoptosis imbalance in β‐cells (Key: FADD, Fas‐associated protein with death domain; mTORC1, mechanistic target of rapamycin complex 1; NIK, NF‐κB‐inducing kinase; TRAF2/3, TNF receptor‐associated factor 2/3; TSC1/2, tuberous sclerosis complex 1/2).

Prostate apoptosis response gene‐4 (PAR‐4) plays an important role in age‐associated conditions such as T2DM through its regulation of programmed cell death. As a tumour‐suppressing protein, PAR‐4 specifically induces apoptosis in malignant cells. Using the yeast two‐hybrid method, Chen Liu et al. discovered a binding interaction between PAR‐4 and TERT. Further investigations confirmed that PAR‐4 and TERT jointly regulate the apoptosis of pancreatic β‐cells in the context of T2DM [55]. In environments characterised by elevated glucose and lipid levels, TERT expression decreases, resulting in reduced interaction with PAR‐4. At the same time, increased PAR‐4 expression activates the endoplasmic reticulum stress pathway in both the plasma membrane and mitochondria. This increase also promotes the translocation of PAR‐4 into the nucleus, where it enhances the activity of the transcription factor NF‐κB, ultimately leading to apoptosis of pancreatic islet β‐cells. The zinc finger motif within PAR‐4 functions as the main interaction site for the serine/threonine kinase Akt, and this segment is essential for regulating PAR‐4‐mediated apoptosis [56, 57]. Akt is a central regulatory enzyme that participates in a range of cellular functions, such as growth, development, programmed cell death and glucose metabolism. Activation of phosphoinositide 3‐kinase (PI3K) leads to the phosphorylation of Akt, generating its active form, known as phosphorylated Akt (p‐Akt). Evidence suggests that p‐Akt promotes the nuclear import of TERT and enhances the stability of the telomerase complex by phosphorylating TERT at serine residue 826 [58]. Furthermore, p‐Akt has been shown to protect pancreatic β‐cells from diabetes‐induced apoptosis and maintain their physiological functionality [59, 60, 61]. Additional research supports a strong interplay between p‐Akt regulation and TERT activity. Downregulation of TERT has been observed to simultaneously hinder the p‐Akt signalling cascade [62]. Also, the lack of TERT leads to gradual telomere attrition, which in turn triggers the expression of the tumour suppressor gene PTEN (phosphatase and tensin homologue deleted on chromosome 10). PTEN acts by dephosphorylating phosphatidylinositol 3,4,5‐trisphosphate (PIP3), thereby suppressing the PI3K/Akt signalling axis and contributing to the development of T2DM [63]. Findings from animal models align with these observations, as mice with β‐cell‐specific deletion of TERT display a 60% decrease in p‐Akt levels and compromised insulin release. Clinical studies have further revealed that certain antidiabetic medications can enhance TERT expression via activation of the p‐Akt pathway, ultimately reducing β‐cell apoptosis in diabetic conditions [64]. The relevant mechanism described above is illustrated in Figure 2.

### 3.3 Glycolysis

TERT enhances glucose uptake and lactate formation while increasing the expression of hexokinase 2 (HK2) [65]. By stabilising c‐Myc, TERT influences the activation or inhibition of several target genes, such as glyceraldehyde‐3‐phosphate dehydrogenase (GAPDH), HK2, and pyruvate kinase M2 (PKM2) [66]. HK2 functions as the initial rate‐limiting enzyme in the glycolytic pathway, facilitating the phosphorylation of glucose to produce D‐glucose 6‐phosphate (G‐6‐P), a key step in glucose metabolism. In hyperglycemic conditions, TERT activity declines, reducing the stability of c‐Myc. This leads to diminished transcription of HK2, thereby directly hindering the production of G‐6‐P [67]. The resulting impairment in G‐6‐P synthesis affects downstream ATP generation, liver glucose release, and glucose utilisation in peripheral tissues, ultimately contributing to increased blood glucose levels [68, 69].

**FIGURE 3:**
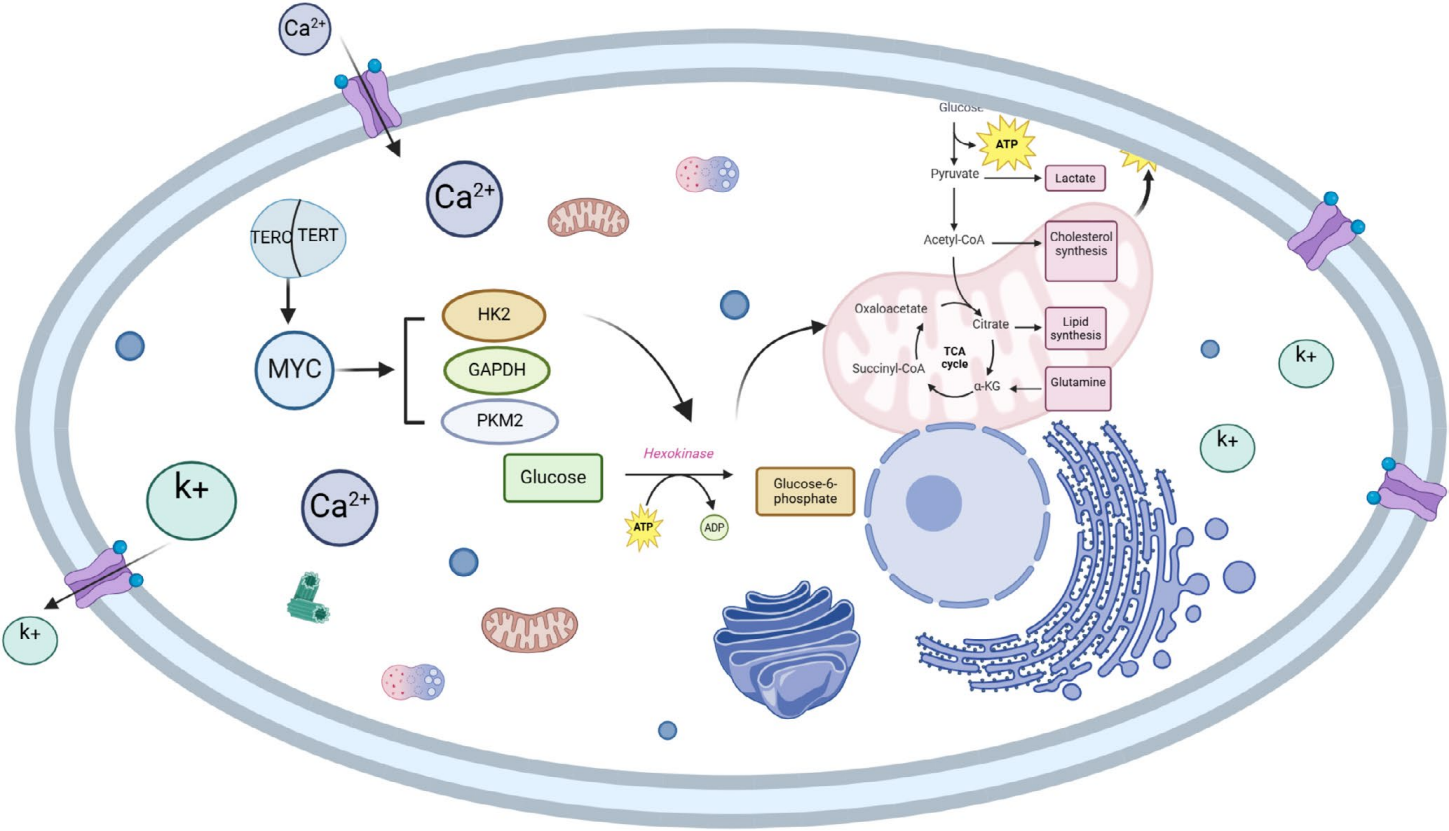
Glycolysis. Disruption of the TERT‐c‐Myc‐glycolysis axis in T2DM. Under hyperglycemia, reduced TERT activity impairs c‐Myc protein stability via the ubiquitin‐proteasome pathway, suppressing transcription of key glycolytic enzymes (e.g., HK2, GAPDH, PKM2). Diminished glycolytic flux leads to: ATP depletion → impaired closure of KATP channels → reduced Ca^2+^ influx → defective insulin granule exocytosis; Pyruvate deficit → insufficient acetyl‐CoA generation → disrupted cytoplasmic cholesterol/fatty acid synthesis. This axis cooperatively mediates β‐cell dysfunction and peripheral tissue insulin resistance in T2DM (Key: GAPDH, glyceraldehyde‐3‐phosphate dehydrogenase; HK2, hexokinase 2; PKM2, pyruvate kinase M2; TCA, Tricarboxylic Acid Cycle).

Additionally, TERT plays a role in regulating the expression of genes associated with inflammation and the activation of inflammasomes by influencing the glycolytic metabolism of innate immune cells, including CD4+ and CD8+ T cells. It can modulate telomerase activity and telomere length either directly or indirectly, thus impacting the development of T2DM [70]. Particularly in a high‐glucose environment, TERT activity decreases. This reduction inhibits the c‐Myc/HK2 pathway, leading to impaired glycolysis in CD4+ and CD8+ T cells and causing metabolic dysfunction in the immune system [71]. It is worth noting that Interleukin‐1 beta (IL‐1β), as one of the important targets for the treatment of T2DM, mainly acts on pancreatic islet β cells, leading to their dysfunction and apoptosis. At the same time, it also participates in the formation of the systemic inflammatory environment and the exacerbation of insulin resistance [72]. A large number of studies have found that the upregulation of IL‐1β is prevalent in T2DM [73, 74]. The resulting deficiency in glycolysis further activates inflammasomes, promoting the release of pro‐inflammatory cytokines such as IL‐1β. This inflammatory reaction subsequently worsens telomere damage through the accumulation of ROS, accelerating the progression of T2DM [75]. The relevant mechanism described above is illustrated in Figure 3.

## 4 Telomere Biology and Targeted Therapeutic Strategies for T2DM

T2DM and its long‐term complications, including macrovascular and microvascular disorders, are strongly influenced by the duration of the disease and the extent of glycemic control [39]. As previously discussed, a hyperglycemic environment can trigger various cellular responses, such as increased oxidative stress, apoptosis, and altered glycolytic function, all of which have a notable impact on telomere length and telomerase function. In the T2DM animal model, the telomerase activity of vascular smooth muscle cells (VSMC) is significantly enhanced and is positively correlated with the degree of cell proliferation [76, 77]. This persistent activation of TERT induced by metabolic stress further drives the pathological over‐proliferation of VSMCs through telomere‐independent transcriptional regulation and mitochondrial antioxidant pathways, ultimately accelerating the progression of diabetic vascular lesions [78]. These observations indicate that telomerase activity could serve as a crucial indicator for evaluating disease progression and predicting outcomes in T2DM, providing a solid theoretical basis for clinical decision‐making and treatment planning. In addition, telomere length also plays a regulatory role in the development and progression of T2DM and its associated complications. Evidence suggests that T2DM patients who develop myocardial infarction tend to have shorter telomeres compared to those without this complication [79]. Murillo‐Ortiz et al. further reported that telomere shortening becomes more severe as the duration of diabetes increases, implying that persistent inflammation may directly accelerate telomere erosion over time [80]. Telomere length has been recognised as a reliable biomarker for assessing the progression of diabetic nephropathy, retinal endothelial aging, and overall cardio‐metabolic health in individuals with T2DM [81]. These results highlight the relationship between telomere attrition and both T2DM and its associated complications, indicating that telomere length could potentially function as a useful biomarker for assessing hyperglycemic and hyperinsulinemic conditions in clinical settings [82].

Based on the theory that telomere dysfunction drives T2DM and its complications, we hypothesised an intervention strategy—pharmacologically activate telomerase to protect the telomere homeostasis of pancreatic islet β‐cells and insulin‐sensitive tissues, delay replicative senescence and simultaneously target and eliminate senescent cells (such as Senolytics) to block the chronic inflammation mediated by the senescence‐associated secretory phenotype, thereby improving the diabetic pathological microenvironment at the root of cellular senescence. Preliminary evidence shows that telomerase activators reversed neurodegeneration in TERT knockout mouse models and eliminated degenerative phenotypes in multiple organs including the testes, spleen, and intestine [83]; after the senescent cell lytic agent (dasatinib plus quercetin) eliminated senescent cells in adipose tissue, the survival rate of mice was increased by 36% [84]. Further development of synergistic drugs (such as metformin combined with Senolytics) may achieve multi‐target alleviation of the disease process by inhibiting oxidative stress, regulating glycolytic flux, and anti‐apoptotic pathways [85].

Recent studies have shown that activating peroxisome proliferator‐activated receptor γ (PPARγ) can boost TERT activity in rats, increase the production of telomere‐related proteins, and lead to improved myocardial contractility [86]. Additionally, emerging evidence suggests that n‐3 fatty acids, often used in the prevention of cardiovascular issues in individuals with T2DM, can also independently stimulate telomerase activity [87]. In addition to influencing telomerase activity, interventions targeting telomere length maintenance offer a promising avenue for T2DM treatment. For example, transplanting amniotic mesenchymal stem cells that have been genetically engineered with the TERT gene into T2DM‐affected rats has been found to markedly reduce blood glucose levels, relieve pancreatic islet damage and effectively alleviate diabetic symptoms [88]. Likewise, TERT‐modified endothelial progenitor cells isolated from rats have shown potential in restoring erectile function in T2DM rats suffering from erectile dysfunction [89]. Moreover, bone marrow‐derived mesenchymal stem cells enhanced with the TERT gene have displayed therapeutic benefits in addressing cardiovascular complications linked to T2DM in animal models [65]. These positive outcomes may stem from elevated secretion of paracrine growth factors and improved antioxidant defences, although the exact mechanisms involved still require further investigation.

The diseases mentioned above appear to be closely linked to age. Although age is a confounding factor for T2DM complications, evidence suggests that patients with T2DM experience accelerated telomere attrition independent of aging [26]. The therapeutic specificity of TERT activation in diabetic complications stems from its modulation of disease‐specific pathological mechanisms rather than exerting a nonspecific anti‐aging effect. In diabetic erectile dysfunction, TERT primarily ameliorates cavernous neurovascular damage induced by hyperglycemia [90]. In diabetic cardiomyopathy, TERT counteracts hyperglycemia and hyperhomocysteinemia‐driven proliferation of VSMCs [91]. These protective effects are distinct from those associated with normal aging‐related tissue degeneration. Future studies should incorporate control groups that account for both age and metabolic disease factors to better disentangle the interplay between metabolic dysregulation and aging.

Researchers have identified TA‐65, a small‐molecule telomerase activator derived from the roots of *Astragalus membranaceus*, which may ameliorate metabolic syndrome by modulating TERT activity in both the nucleus and mitochondria. Evidence from existing studies indicates that TA‐65 improves metabolic parameters such as fasting blood glucose and insulin levels in human randomised controlled trials (RCTs, *n* = 34) [92]. In animal studies (*n* = 48), TA‐65 has been shown to extend the shortest telomeres [93]. However, these findings are constrained by limited sample sizes, and the precise molecular target through which TA‐65 activates TERT remains unclear. Therefore, there is an urgent need to integrate structural biology approaches with dynamic monitoring techniques to elucidate the exact interaction mechanism between TA‐65 and TERT.

## 5 Discussion

In conclusion, through an in‐depth exploration of the roles of telomeres and telomerase in T2DM and its complications, a significant association between T2DM and telomere attrition has been identified. This relationship is driven by key factors such as mitochondrial dysfunction, chronic inflammation, dysregulated glycolysis, and oxidative stress, forming a complex bidirectional reinforcement mechanism rather than a simple linear causality. Initially, the hallmark hyperglycemia of T2DM promotes the accumulation of ROS via the formation of advanced glycation end products (AGEs) and activation of the polyol pathway [94]. Concurrently, the persistent inflammatory state—characterised by elevated levels of pro‐inflammatory cytokines such as TNF‐α and IL‐6—further intensifies oxidative stress [95]. Together, these conditions trigger the DDR, suppress telomerase activity through pathways including p53‐p21, and accelerate telomere shortening [96]. Secondly, when telomeres shorten to a critical length or undergo accelerated attrition, they induce senescence and apoptosis in pancreatic β‐cells, evidenced by reduced proliferative capacity, disrupted insulin secretion signalling pathways, and increased cell death, ultimately impairing insulin production [97]. Consequently, metabolic stress drives telomere attrition, while shortened telomeres exacerbate metabolic dysfunction by promoting β‐cell failure and increasing insulin resistance in peripheral tissues. This reciprocal interaction creates a self‐reinforcing cycle, potentially establishing a positive feedback loop: ‘metabolic stress → telomere shortening → cellular dysfunction → metabolic decompensation → heightened metabolic stress’.

There are several limitations in this study. For instance, the regulatory mechanisms underlying telomere length and telomerase activity are highly complex, and the association between telomere attrition and type 2 diabetes mellitus (T2DM) involves multiple interrelated factors across various biological levels. The scope of this paper covers only a subset of this intricate network and therefore lacks comprehensive and in‐depth analysis. Nevertheless, given the critical role of telomeres in human disease pathogenesis, future research should aim to further elucidate the specific contributions of telomeres and telomerase to T2DM development and its underlying mechanisms. Such investigations may facilitate the discovery of novel therapeutic strategies targeting telomere maintenance, offering promising avenues for the treatment of T2DM and related metabolic disorders.

## Author Contributions

Hongye Cao: conceptualisation, investigation, visualisation. Hongye Cao: writing – original draft. Hongye Cao: writing – review and editing. Haixia Feng: data curation. Zhen Shen: formal analysis. Yanqin Huang: funding acquisition. Yanqin Huang: project administration. Yanqin Huang: resources. Yanqin Huang: supervision.

## Funding

The research was supported by National Natural Science Foundation of China (81974562), Shandong Provincial Natural Science Foundation of China (ZR2024MH245), Shandong Taishan scholar project (tsqn202211354), Shandong Key Laboratory of Traditional Chinese Medicine efficacy and Mechanism (PKL2024C23), and Construction and efficacy evaluation of an intelligent innovation platform for weight management utilizing traditional Chinese medicine (2025CXPT147).

## Ethics Statement

All authors are familiar with the contents of the final draft and take responsibility for the authenticity of the data used in the paper. This manuscript is original and has not been previously published, nor has it been simultaneously submitted to any other journal.

## Consent

The manuscript has been approved by all authors for publication.

## Conflicts of Interest

The authors declare no conflicts of interest.

## Data Availability

Data availability is not applicable to this article as no new data were created or analysed in this study.
